# R.ROSETTA: an interpretable machine learning framework

**DOI:** 10.1186/s12859-021-04049-z

**Published:** 2021-03-06

**Authors:** Mateusz Garbulowski, Klev Diamanti, Karolina Smolińska, Nicholas Baltzer, Patricia Stoll, Susanne Bornelöv, Aleksander Øhrn, Lars Feuk, Jan Komorowski

**Affiliations:** 1 Department of Cell and Molecular Biology, Uppsala University, Uppsala, Sweden; 2 Department of Immunology, Genetics and Pathology, Uppsala University, Uppsala, Sweden; 3 Department of Research, Cancer Registry of Norway, Oslo, Norway; 4 Department of Biosystems Science and Engineering, ETH Zurich, Zurich, Switzerland; 5 Cancer Research UK Cambridge Institute, University of Cambridge, Cambridge, UK; 6 Department of Informatics, University of Oslo, Oslo, Norway; 7 Swedish Collegium for Advanced Study, Uppsala, Sweden; 8 Institute of Computer Science, Polish Academy of Sciences, Warsaw, Poland; 9 Washington National Primate Research Center, Seattle, WA USA

**Keywords:** Transcriptomics, Interpretable machine learning, Big data, Rough sets, Rule-based classification, R package

## Abstract

**Background:**

Machine learning involves strategies and algorithms that may assist bioinformatics analyses in terms of data mining and knowledge discovery. In several applications, viz. in Life Sciences, it is often more important to understand how a prediction was obtained rather than knowing what prediction was made. To this end so-called interpretable machine learning has been recently advocated. In this study, we implemented an interpretable machine learning package based on the rough set theory. An important aim of our work was provision of statistical properties of the models and their components.

**Results:**

We present the R.ROSETTA package, which is an R wrapper of ROSETTA framework. The original ROSETTA functions have been improved and adapted to the R programming environment. The package allows for building and analyzing non-linear interpretable machine learning models. R.ROSETTA gathers combinatorial statistics via rule-based modelling for accessible and transparent results, well-suited for adoption within the greater scientific community. The package also provides statistics and visualization tools that facilitate minimization of analysis bias and noise. The R.ROSETTA package is freely available at https://github.com/komorowskilab/R.ROSETTA. To illustrate the usage of the package, we applied it to a transcriptome dataset from an autism case–control study. Our tool provided hypotheses for potential co-predictive mechanisms among features that discerned phenotype classes. These co-predictors represented neurodevelopmental and autism-related genes.

**Conclusions:**

R.ROSETTA provides new insights for interpretable machine learning analyses and knowledge-based systems. We demonstrated that our package facilitated detection of dependencies for autism-related genes. Although the sample application of R.ROSETTA illustrates transcriptome data analysis, the package can be used to analyze any data organized in decision tables.

**Supplementary Information:**

The online version contains supplementary material available at 10.1186/s12859-021-04049-z.

## Background

Machine learning approaches aim at recognizing patterns and extracting knowledge from complex data. In this work, we aim at supporting the knowledge-based data mining with an interpretable machine learning framework [1, 2]. Recently, understanding the complex machine learning classifiers that explain their output is a highly important topic [3]. Here, we implemented an R package for a non-linear interpretable machine learning analysis that is based on rough set theory [4]. Moreover, we enriched our tool with basic statistical measurements that is a unique development with comparison to current state-of-the-art tools. At the end of this section, we also briefly introduce the mathematical theory behind rough sets. For a complete presentation of rough sets the reader is recommended to consult the tutorial [5] or other literature [1, 4, 6–8].

Classification models are trained on labeled objects that are a priori assigned to them. The universal input structure for machine learning analyses is a decision table or decision system [9–11]. This concept is similar to feature matrix that is well-known in image analysis [12, 13]. These structures organize data in a table that contains a finite set of features, alternatively called attributes or variables. However, decision tables are adapted for supervised learning. Specifically, a set of objects, also called examples or samples, is labelled with a decision or outcome variable. Decision system is defined as $\mathcal{D}=\left(U,A\cup \left\{d\right\}\right)$ where $U$ is a non-empty finite set of objects called universe, $A$ is a non-empty finite set of features and $d$ is the decision such that $d\notin A$ [5]. Importantly, most of the omics datasets can be represented as decision tables, and machine learning analysis can be applied to a variety of problems such as, for instance, case–control discrimination. When analyzing ill-defined decision tables, i.e. tables where $\left|A\right|\gg \left|U\right|$, an appropriate feature selection step is necessary prior to the machine learning analysis [14–16]. The main goal of feature selection is to reduce the dimensionality to the features that are relevant to outcome. Thus, it is recommended to consider feature selection as a standard step prior to the machine learning analysis, especially for big omics datasets such as transcriptome data.

**Fig. 1:**
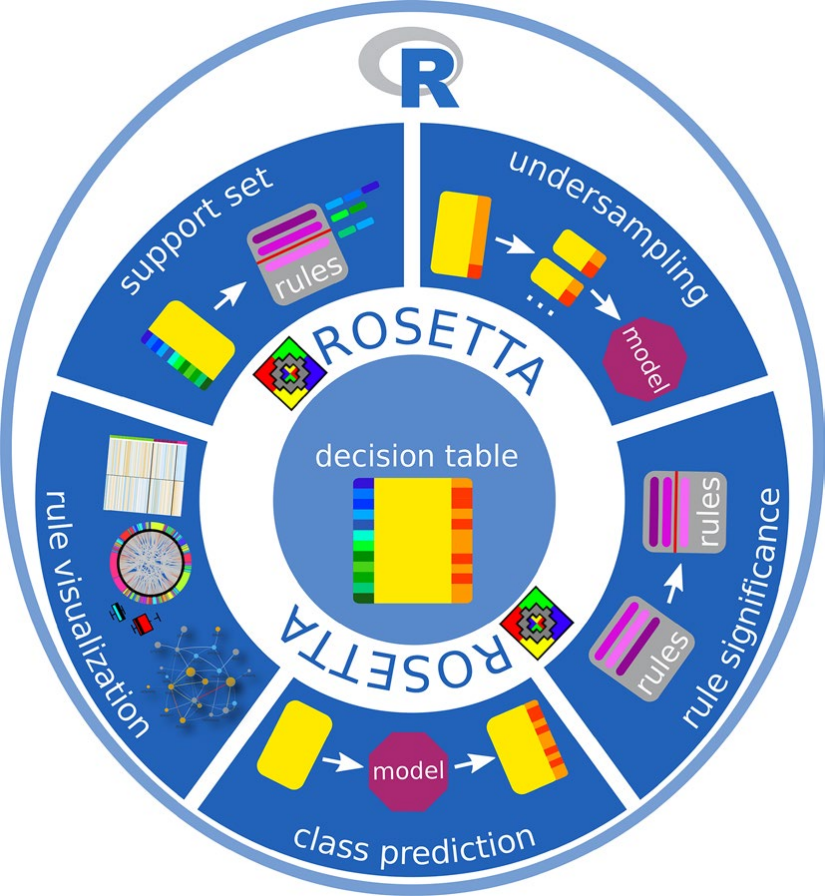
Overview of R.ROSETTA and the major components that were implemented to enhance the ROSETTA functionality. (Icon with R logo included in this figure was released by the R Foundation under the CC BY-SA 4.0 license)

The ROSETTA software is an implementation of a framework for rough set classification [17]. It was implemented in C++ as a graphical user interface (GUI) and command line version. ROSETTA has been successfully applied in various studies to model biomedical problems [8, 18, 19]. Here, we present a more accessible and flexible implementation of ROSETTA that was used as the core program of the R package. R.ROSETTA substantially extends the functionality of the existing software towards analyzing complex and ill-defined bioinformatics datasets. Among others, we have implemented functions such as undersampling, estimation of rule-statistical significance, prediction of classes, merging of models, retrieval of support sets and various approaches to model visualization (Fig. 1). To the best of our knowledge, there is no framework that allows for such broad analysis of interpretable classification models. Overall, rough set-based algorithms proved successful in knowledge and pattern discovery [20–22]. Here, we illustrated the functionality of R.ROSETTA by exploring rule-based models for synthetically generated datasets and transcriptomic dataset for patients with and without autism, hereafter called the autism-control dataset.

### Rough sets

Rough set theory has become an inherent part of interpretable machine learning. In recent years, the rough sets methodology has been widely applied to various scientific areas, e.g. [23–25]. It supports artificial intelligence research in classification, knowledge discovery, data mining and pattern recognition [4, 7]. One of the most important properties of rough sets is the discovery of patterns from complex and imperfect data [26, 27]. The principal assumption of rough sets is that each object $x$ such that$x\in X$, where$X\subseteq U$, is represented by an information vector. In particular, objects identified with the same information vectors are indiscernible. Let $\mathcal{D}=\left(U,A\cup \left\{d\right\}\right)$ be a decision system. For any subset of features $B\subseteq A$ there is an equivalence relation${IND}_{\mathcal{D}}\left(B\right)$, called $B$-indiscernibility relation [5]:

1 $${IND}_{\mathcal{D}}\left(B\right)= \left\{\left(x,x\mathrm{^{\prime}}\right)\in {U}^{2} : \forall a\in B a\left(x\right)=a\left(x\mathrm{^{\prime}}\right)\right\}$$

where $\left(x,x\mathrm{^{\prime}}\right)$ are objects that are indiscernible from each other by features from $B$ if $\left(x,x\mathrm{^{\prime}}\right)\in {IND}_{\mathcal{D}}\left(B\right)$.

Consider three subsets of features: ${B}_{1}=\left\{{gene}_{1}\right\}$, ${B}_{2}=\left\{{gene}_{2}\right\}$ and ${B}_{3}=\left\{{gene}_{1},{gene}_{2}\right\}$ for the decision system in Table 1. Each of the indiscernibility relations defines a partition of $U$ (1) ${IND}_{\mathcal{D}}\left({B}_{1}\right)= \left\{\left\{{x}_{1},{x}_{2},{x}_{3}\right\},\left\{{x}_{4},{x}_{5}\right\}\right\}$ (2) ${IND}_{\mathcal{D}}\left({B}_{2}\right)= \left\{\left\{{x}_{1},{x}_{3},{x}_{5}\right\},\left\{{x}_{2},{x}_{4}\right\}\right\}$ and (3) ${IND}_{\mathcal{D}}\left({B}_{3}\right)= \left\{\left\{{x}_{1},{x}_{3}\right\},\left\{{x}_{2}\right\},\left\{{x}_{4}\right\},\left\{{x}_{5}\right\}\right\}$. For example, using ${B}_{1}$, objects $\left\{{x}_{1},{x}_{2},{x}_{3}\right\}$ are indiscernible and thus belong to the same equivalence class [5].

**Table 1:** An example decision table $\mathcal{D}=\left(U,A\cup \left\{d\right\}\right)$ where $A$ is a set of two genes, $U$ is a set of five objects ${x}_{1}$, …, ${x}_{5}$ and $d$ is case or control diagnosis. The values in $U$ are discrete gene expression levels “up” or “down”

Object	${gene}_{1}$	${gene}_{2}$	$diagnosis$
${x}_{1}$	Up	Up	Case
${x}_{2}$	Up	Down	Case
${x}_{3}$	Up	Up	Control
${x}_{4}$	Down	Down	Control
${x}_{5}$	Down	Up	Case

Let us consider decision system $\mathcal{D}$ with a subset of features $B\subseteq A$ and subset of objects$X\subseteq U$. We can then approximate $X$ using features from $B$ by constructing the so-called $B$-lower and $B$-upper approximations of $B$ expressed as $\underset{\_}{B}X$ and $\overline{B}X$, respectively, where $\underset{\_}{B}X=\left\{x : {\left[x\right]}_{B}\subseteq X\right\}$ and $\overline{B}X=\left\{x : {\left[x\right]}_{B}\cap X\ne \mathrm{\varnothing }\right\}$. The $B$-lower approximation contains objects that certainly belong to $X$ and the $B$-upper approximation contains objects that may belong to$X$. The set ${Bnd}_{B}\left(X\right)=\overline{B}X -\underset{\_}{B}X$ is called $B$-boundary region of $X$. The set $X$ is called rough if ${Bnd}_{B}\left(X\right)\ne \varnothing$ and crisp otherwise. The objects that certainly do not belong to *X* are in the $B$-outside region and their set is defined as$U-\overline{B}X$. For example, if$X=\left\{x : diagnosis\left(x\right)=case\right\}$, as in Table 1, then the approximation regions are$\underset{\_}{A}X=\left\{{x}_{2},{x}_{5}\right\}$,$\overline{A}X=\left\{{{x}_{1},x}_{2},{x}_{3},{x}_{5}\right\}$, $B{nd}_{A}\left(X\right)=\left\{{x}_{1},{x}_{3}\right\}$ and$U-\overline{A}X=\left\{{x}_{4}\right\}$.

For such example (Table 2) we can define another table called decision-relative discernibility matrix $M$ as shown in the literature [5, 28]. From $M$ we can construct a discernibility function ${f}_{\mathcal{D}}M\left(A\right)$ that is a Boolean [29] function in a conjunctive normal form of disjunctive literals where the literals are the names of features that discern in a pair-wise fashion equivalence classes with different decisions. For example, $\left({g}_{1}\vee {g}_{3}\vee rf\right)$ discerns between ${q}_{1}$ and ${q}_{4}$. Next, the Boolean formula is minimized and called a reduct. The discernibility function for our decision system is ${f}_{\mathcal{D}}M\left(A\right)=\left({g}_{1}\vee {g}_{3}\vee rf\right)\left({g}_{2}\vee {g}_{3}\vee rf\right)\left({g}_{3}\vee rf\right)\left({g}_{3}\vee rf\right)\left({{g}_{1}\vee g}_{2}\vee {g}_{3}\vee rf\right)\left({g}_{1}\vee {g}_{3}\vee rf\right)\left({g}_{2}\vee rf\right)\left({g}_{1}\vee {g}_{2}\vee rf\right)\left({g}_{1}\vee {g}_{2}\vee rf\right)\left({{g}_{1}\vee g}_{2}\vee {g}_{3}\right)\left({g}_{2}\vee {g}_{3}\right)\left({{g}_{1}\vee g}_{2}\right)\left({g}_{2}\right)\left({g}_{2}\right)\left({g}_{2}\vee {g}_{3}\vee rf\right)\left({{g}_{1}\vee g}_{2}\vee {g}_{3}\vee rf\right)\left({{g}_{1}\vee g}_{2}\vee {g}_{3}\vee rf\right)\left({g}_{2}\vee {g}_{3}\vee rf\right)\left({g}_{2}\vee {g}_{3}\vee rf\right)\left({g}_{2}\right)\left({{g}_{1}\vee g}_{2}\right)$ that after a simplification results in two reducts ${f}_{\mathcal{D}}M\left(A\right)=\left({g}_{2}\wedge rf\right)\vee \left({g}_{2}\wedge {g}_{3}\right)$. From the construction it follows that the reducts have the same discernibility as the full set of features. This study investigates two algorithms of computing reducts, called reducers, the Johnson reducer [30] which is a deterministic greedy algorithm, and the Genetic reducer [31] which is a stochastic method based on the theory of genetic algorithms. The reader may notice that this process is a form of feature selection. Finally, each reduct gives rise to rules by overlaying it over all objects in $\mathcal{D}$ (Table 2). For example, the first reduct $\left({g}_{2}\wedge rf\right)$ and the equivalence class ${q}_{1}$ give a rule $\mathrm{IF }\,{g}_{2}=\mathrm{low}\, \mathrm{AND} \, rf=\mathrm{yes}\, \mathrm{THEN }\,diagnosis=\mathrm{autism}$. The IF-part of the rule consists of conjuncts and is called the condition (or predecessor, or left-hand side) of the rule and the THEN-part is a conclusion (or successor, or right-hand side). Importantly, rules can have an arbitrary, but finite number of conjuncts.

**Table 2:** An example generalized decision table $\mathcal{D}=\left(U,A\cup \left\{d\right\}\right)$ for case–control study of autism, where $A$ is a set of three genes $\left\{{g}_{1},{g}_{2},{g}_{3}\right\}$ and a risk factor $\left\{rf\right\}$, and $U$ is a set of objects that belong to equivalence classes ${[q}_{1}], \dots , [{q}_{8}]$. The values in $U$ are discrete gene expression levels “low”, “medium” or “high” and a presence of undefined risk factor “yes” or “no”. For simplicity we omit the brackets in the notation in the table. For the equivalence classes ${q}_{4}$ and ${q}_{5}$ both diagnoses are written since some of the indiscernible objects belong to the boundary region

Equivalence class	${g}_{1}$	${g}_{2}$	${g}_{3}$	$rf$	$diagnosis$
${q}_{1}$	Low	Low	Medium	Yes	Autism
${q}_{2}$	Medium	Medium	Medium	Yes	Autism
${q}_{3}$	Medium	Low	Medium	Yes	Autism
${q}_{4}$	Medium	Low	High	No	Autism or control
${q}_{5}$	Low	Low	High	No	Autism or control
${q}_{6}$	Low	High	Medium	No	Control
${q}_{7}$	Medium	High	Medium	Yes	Control
${q}_{8}$	Medium	High	High	No	Control

### Numerical characterization of rules

Rules are frequently described with measurements of support, coverage and accuracy. The rule support represents the number of objects that fulfill the rule conditions. Left-hand side support (LHS support) is the number of objects that satisfy the rule conjuncts i.e. IF-part of the rule. Right-hand side support (RHS support) is the number of the LHS objects of the respective classes i.e. of the THEN-part of the rule. The rule coverage can be explicitly determined from the LHS or RHS support as a percentage of objects contributing to the rule. We discern between RHS and LHS coverage:

2 $${coverage}_{RHS}\text{(}rule\text{) }\text{=} \, \frac{{support}_{RHS}(rule)}{{n}_{d}}$$

3 $${coverage}_{LHS}\text{(}rule\text{) }\text{=} \, \frac{{support}_{LHS}(rule)}{{n}_{d}}$$

where ${n}_{d}$ is the total number of objects from $U$ for a decision class $d$ defined by the rule. Accuracy of the rule represents its predictive strength that is computed based on the support values. Specifically, accuracy for a rule is calculated as:

4 $$accuracy\text{(}rule\text{) }\text{=} \, \frac{{support}_{RHS}(rule)}{{support}_{LHS}(rule)}$$

### Johnson reducer

The Johnson reducer belongs to the family of greedy algorithms. For the decision table $\mathcal{D}=\left(U,A\cup \left\{d\right\}\right)$, the main aim of the Johnson algorithm is to find a feature $a\in A$ that discerns the highest number of object pairs [32]. Computing reducts with Johnson approach has time complexity $O(k\bullet {m}^{2}\bullet \left|R\right|)$, where $k$ is the number of features, $m$ is the number of objects and $R$ is the computed reduct [32]. The Johnson algorithm for computing a single reduct is expressed as follows [33]: (1) Let $R=\varnothing .$(2) Let ${a}_{max}\in A$ be the feature that maximizes $\sum w(S)$ where $w(S)$ denotes a weight for subsets $S\subseteq \mathcal{S}$ for set $\mathcal{S}$ obtained from discernibility matrix. The sum is taken over all $S$ from $\mathcal{S}$ that contain ${a}_{max}$. (3) Add ${a}_{max}$ to $R$. (4) Remove all $S$ from $\mathcal{S}$ that contain ${a}_{max}$. (5) If $\mathcal{S}=\varnothing$ return $R$. Otherwise, go to step 2.

### Genetic reducer

The genetic algorithm is based on Darwin’s theory of natural selection [31]. This is a heuristic algorithm for function optimization that follows the “survival of the fittest” idea [34]. It simulates the selection mechanism with a fitness function $f$ [33, 34] that rewards hitting sets$B$:

5 $$f\left(B\right)=\left(1-\alpha \right)\times \frac{cost\left(A\right)-cost\left(B\right)}{cost\left(A\right)}+\alpha \times min\left\{\varepsilon , \frac{\left|\left[S\subseteq \mathcal{S} : S\cap B\ne \varnothing \right]\right|}{\left|\mathcal{S}\right|}\right\}$$

where $B$ are hitting sets such that $B\subseteq A$ found through the search by the fitness function, $\mathcal{S}$ is a set obtained from discernibility matrix, $\alpha$ is a control parameter for weighting between subset cost and hitting fraction, and $\varepsilon$ is the degree of approximation, i.e. hitting sets $B$ that have a hitting fraction at least $\varepsilon$ are kept in the list. For the Genetic reducer, the most time-consuming part is the fitness computation. The time complexity for the fitness function is $O(k\bullet {m}^{2})$ [31]. A more detailed description of applying genetic algorithm for estimating reducts can be found in [31].

## Implementation

R.ROSETTA was implemented under R [35] version 3.6.0 and the open-source package is available on GitHub (https://github.com/komorowskilab/R.ROSETTA). The R.ROSETTA package is a wrapper (Additional file 1: Package architecture) of the command line version of the ROSETTA system [17, 36]. In contrast to ROSETTA, R.ROSETTA is an R package with multiple additional functionalities (Fig. 1). The following sections cover a detailed description of the new functions.

### Undersampling

Class imbalance issue may lead to biased performance of the machine learning models [37, 38]. Ideally, each decision class shall contain approximately the same number of objects. To tackle this, we suggested to randomly sample a sufficient number of times the majority class without replacement in order to achieve an equal representation of classes. This approach of balancing the data is generally known as undersampling [37].

To build a balanced rule-based model, we have implemented an option that divides the dataset into subsets of equal sizes by undersampling the larger sets. By default, we require each object to be selected at least once, although the user can specify a custom number of sampled sets, as well as a custom size for each set. Classification models for each undersampled set are merged into a single model that consists of unique rules from each classifier. The overall accuracy of the model is estimated as the average value of the sub-models. Finally, the statistics of the merged rule-set shall be recalculated on the original training set using the function *recalculateRules*. Herein, the recalculation procedure compares each rule from trained model to the features from original data and calculates adjusted statistics.

### Rule significance estimation

The *P* value is a standard measure of statistical significance in biomedical studies. Here, we introduced *P* value estimation as a quality measure for the rules. Classification models generated by R.ROSETTA consist of sets of varying number of rules estimated by different algorithms. In the case of the Johnson algorithm, this set contains a manageable number of rules (Table 3, Additional file 1: Table S1), while in the case of the Genetic algorithm this set can be considerably larger (Additional file 1: Tables S1, S2). In both cases, supervised pruning of rules from the models would not heavily affect the overall performance of the classifier. To better assess the quality of each rule we assume a hypergeometric distribution to compute *P* values [39] followed by multiple testing correction. The hypergeometric distribution estimates the representation of the rule support against the total number of objects. When estimating the *P* value for a rule, the hypergeometric distribution is adapted to the rule concepts:

6 $$P\left(X=x\right)=\frac{\left(\genfrac{}{}{0pt}{}{{n}_{d}}{x}\right)\left(\genfrac{}{}{0pt}{}{{n}_{o}}{y-x}\right)}{\left(\genfrac{}{}{0pt}{}{N}{y}\right)}$$

where $x$ is the RHS support of the rule, $y$ is the LHS support of the rule, ${n}_{d}$ is the total number of objects matching the decision class $d$ defined by the rule,${n}_{o}$ is the number of objects for the decision class(es) opposite to the given rule and $N$ is the total number of objects. Models enriched with rule *P* values can be pruned based on significance levels to illustrate the essential co-predictive mechanisms among the features. Additionally, the user may apply multiple testing correction. Herein, we used rigorous Bonferroni correction in order to protect from type I error and to account for the large number of rules generated by the Genetic reducer. To compare both reducers upon the same assumptions, Bonferroni correction was used also for rules generated with the Johnson reducer. However, this parameter can be tuned in R.ROSETTA for a less stringent correction that can be more adequate for models generated with the Johnson reducer only. We also implemented additional model-tuning statistical metrics for rules including risk ratio, risk ratio *P* value and risk ratio confidence intervals that are estimated with the R package fmsb [40]. Full set of statistical measurements is included in the output of the R.ROSETTA model.

**Table 3:** Performance evaluation of rules for the Johnson reduction method with undersampling. The average statistic values of rule support and accuracy are presented in the table. For the rule statistics, the most significant co-predictors (Bonferroni-adjusted *P* ≤ 0.05) were selected

Class	Control		Autism	
Total number of rules	207		194	
Rule statistics	Basic	Recalculated	Basic	Recalculated
Number of rules (*P* ≤ 0.05)	150	89	128	94
LHS support	13	18	13	16
RHS support	13	17	13	14
Accuracy	0.97	0.94	0.98	0.85
Top co-predictors	PPOX, NCS1	MAP7, NCKAP5L	RHPN1, ZFP36L2	NCS1, CSTB

### Vote normalization in the class prediction

Rule-based models allow straightforward class prediction of unseen data using voting. Every object from the provided dataset is fed into the pre-trained machine-learning model and the number of rules for which their LHS is satisfied are counted in. In the final step, the votes from all rules are collected for each individual object. Typically, an object is assigned to the class with the majority of votes. However, for some models an imbalanced number of rules for each decision class over another may have been generated. For example, Johnson model generated more rules for control than the autism class (Table 3). This imbalance may impact the voting procedure. For such cases, we proposed adjusting for the rule-imbalance by normalizing the result of voting. Herein, vote counts represent the number of rules from trained model that match features and their discrete values from an external test set. We implemented various vote normalization methods in R.ROSETTA. Vote normalization can be performed by dividing the number of counted votes by its mean, median, maximum, total number of rules or square root of the sum of squares. We compared the performance of these methods in (Additional file 1: Table S3).

### Rule-based model visualization

The model transparency is an essential feature that allows visualization of co-predictive mechanisms in a local (single rule) and global (whole model) scale. The package provides several ways for visualizing single rules, including boxplots and heatmaps (Additional file 1: Figs. S1d, S2) that illustrate the continuous levels of each feature of the selected rule for each object. Such rule-oriented visualizations gather the objects into those that belong to the support set for the given class, those that do not belong to the support set for the given class and the remaining objects for the other classes. Such graphic representations can assist towards the interpretation of individual rules of interest and visualization of interactions with respect to their continuous values.

A more holistic approach displays the entire model as an interaction network [41]. The R.ROSETTA package allows exporting the rules in a specific format which is suitable with rule visualization software such as Ciruvis [42] or VisuNet (Additional file 1: Fig. S1b) [43, 44]. Such model can be pruned to display only the most relevant co-predictive features and their levels. These approaches provide a different point of view on the interpretation of machine learning models that allow discovering known proof-of-concept and novel co-predictive mechanisms among features [45, 46].

### Recapture of support sets

R.ROSETTA is able to retrieve support sets that represent the contribution of objects to rules (Additional file 1: Figs. S1d, S2). As a result, each rule is characterized by a set of objects that fulfill the LHS or RHS support. For example, in case of the gene expression data, gene co-predictors will be represented with the list of corresponding samples (patients). There are several advantages into knowing this information for the corresponding objects. Support sets contribute to uncovering objects whose levels of features might have shared patterns. Such sets may be further investigated to uncover specific subsets within decision classes. Moreover, non-significant support sets allow detecting objects that may potentially introduce a bias to the model and might be excluded from the analysis.

### Synthetic data

To evaluate rule-based modelling with R.ROSETTA, we implemented a function to create synthetic data. The synthetic dataset can be generated with a predefined number of features, number of objects and proportion of classes. Additionally, the user may choose between continuous and discrete data. The synthetic data structure is formulated as a decision table that follows the description in the introduction. A synthetic dataset is constructed from the transformation of randomly generated features computed from a normal distribution. In this approach, the randomly generated features are multiplied by the Cholesky decomposition of positive-definite covariance matrix [47]. The Cholesky decomposition *D* of the matrix *L* is calculated as:

7 $$D=L{L}^{T}$$

where *L* is a lower triangular covariance matrix, and *L*^*T*^ is conjugate transpose of *L*.

## Results and discussion

### Benchmarking

**Fig. 2:**
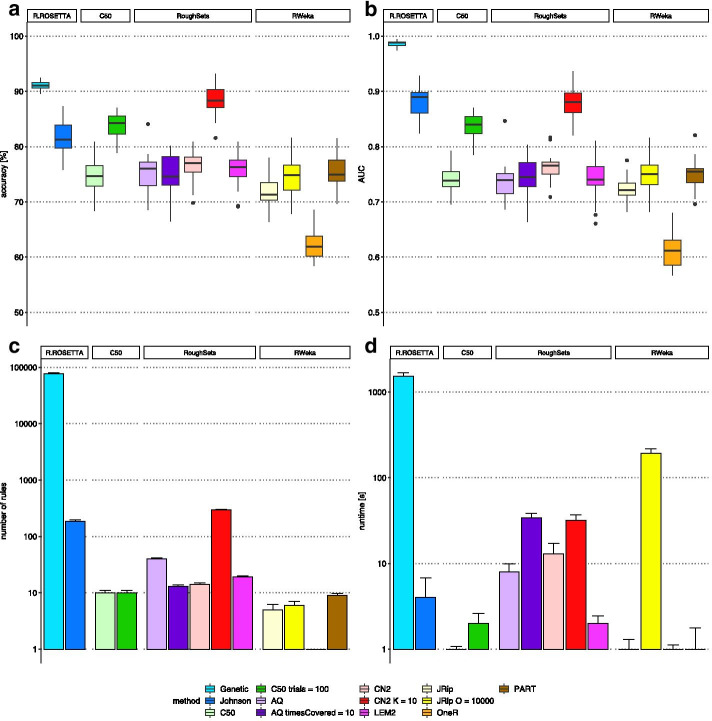
Benchmarking the R packages for rule-based machine learning applied onto the autism-control dataset. The packages were evaluated with various methods for default and tuned parameters. For the R.ROSETTA package, Johnson reducer (Johnson) and Genetic reducer (Genetic) were used. For the package C50, the C5.0 method (C50) was used. For the package RoughSets, Learning from Examples Module (LEM2), CN2 rule induction (CN2) and Quasi-optimal covering Algorithm (AQ) were used. For the package RWeka Repeated Incremental Pruning to Produce Error Reduction (JRip), 1-rule classifier (OneR) and partial decision trees-based (PART). Several methods were tuned for the number of boosting iterations (*trials*), times covering objects by rules (*tc*), algorithm complexity (*K*) and number of optimizations (*O*). Other methods were evaluated with default parameters. The results of benchmarking are presented for **a** accuracy distribution of classifiers, **b** ROC AUC distribution of classifiers, **c** number of estimated rules (logarithmic scale) and **d** average runtime of the algorithms (logarithmic scale). Two standard deviations were marked above each bar. The time was measured from inputting a decision table to receiving a model

We benchmarked R.ROSETTA against three other R packages that perform rule-based machine learning including C50 [48], RoughSets [49] and RWeka [50] (Additional file 1: Benchmarking, Table S4). Using the autism-control dataset, we compared the efficacy of the classification algorithms by measuring the accuracy, the area under the ROC curve (AUC), the running time and the total number of rules (Fig. 2). To perform compatible benchmarking across algorithms, we standardized the classification procedure for equal frequency discretization and tenfold cross validation (CV). Additionally, to account for the stochasticity introduced by sampling in CV, each algorithm was executed 20 times with different seed values.

Even though R.ROSETTA produced one of the highest quality models (Fig. 2a, b), its runtime, especially for the genetic algorithm, was higher than of the other algorithms (Fig. 2d). We highlight that computing single reduct is linear in time while finding all minimal reducts is an NP-hard problem [5, 34]. In contrast to other systems, R.ROSETTA computes all minimal reducts. We believe this is an important feature since biological systems are robust and usually have alternative ways of achieving the outcome. Clearly, as a consequence of estimating multiple reducts, R.ROSETTA algorithms tend to produce more rules in comparison to other methods (Fig. 2c). It is then natural to remove the weakest rules and obtain simpler and more interpretable models. To this end, we suggest to prune the set of rules using the quality measurements such as, for instance, support, coverage or *P* value. Furthermore, we observed that the surveyed packages do not provide straightforward quality-statistic metrics for the model and the rules. The R.ROSETTA package includes a variety of quality and statistical indicators for models (accuracy, AUC etc.) and rules (support, *P* value, risk ratio etc.) in an effortlessly and R-friendly inspectable output. Notably, the evaluated packages do not include newly implemented R.ROSETTA features such as undersampling, support sets retrieval and rule-based model visualizations.

In addition, we benchmarked R.ROSETTA against methods based on decision trees, which is a concept closely related to the rule-based systems [1] (Additional file 1: Fig. S3). Both are considered as highly interpretable approaches that are able to capture non-linear dependencies among features. However, the main advantage of rough sets over the decision trees is an improved stability of models [51, 52]. To compare the performance of R.ROSETTA with tree-based methods, we investigated regression trees from package rpart [53], bagging from package ipred [54], random forest from package randomForest [55] and generalized boosted regression models from package GBM [56]. The evaluation has been performed with the autism-control dataset using the same discretization and CV approach as for the rule-based packages. The results showed that R.ROSETTA performance is, in terms of accuracy, similar to tree-based methods (Additional file 1: Fig. S3a). However, both R.ROSETTA reduction methods had the highest median AUCs among all tested approaches (Additional file 1: Fig. S3b). Moreover, increasing the number of trees or replications for the tree-based methods resulted in a time complexity similar to the Genetic reducer, although without outperforming the rule-based classifiers (Additional file 1: Fig. S3c). Majority of benchmarked methods showed that the dataset is well-predictable. However, we emphasize that diverse datasets can perform in various ways. Furthermore, the choice of feature selection method can also play a major role in the final performance.

### Sample application of R.ROSETTA on transcriptome data

To illustrate R.ROSETTA in a bioinformatics context, we applied the tool to a sample transcriptome analysis task. We examined gene expression levels of 82 male children with autism and 64 healthy male children (control) (Additional file 1: Table S5) downloaded from the GEO repository (GSE25507) [57]. The expression of 54,678 genes was measured from the peripheral blood material with the Affymetrix Human Genome U133 Plus 2.0 array. Previously, it has been reported that blood can be used as effectively as brain for transcriptomic studies of autism [58, 59]. Importantly, while obtaining the samples, blood is less invasive tissue than brain [58]. Other studies suggested that blood–brain barrier and immune system are altered in subjects with autism [60, 61]. The dataset was preprocessed (Additional file 1: Data preprocessing) and corrected for the effect of age (Additional file 1: Fig. S4). The decision table was ill-defined with the number of genes being much larger than the number of samples. To handle such high-dimensional data, we employed the Fast Correlation-Based Filter (FCBF) [62] that is a classifier-independent method of feature filtration. FCBF belongs to the group of filter-based methods, thus can be used prior to the learning processes [63, 64]. Furthermore, we favored the FCBF method as an algorithm with low time complexity and operating on pre-discretized data (Additional file 1: Feature selection, Table S6). The final decision table was reduced to 35 genes (Additional file 1: Table S7), which allowed us to generate classifiers with a reasonably low time complexity.

**Fig. 3:**
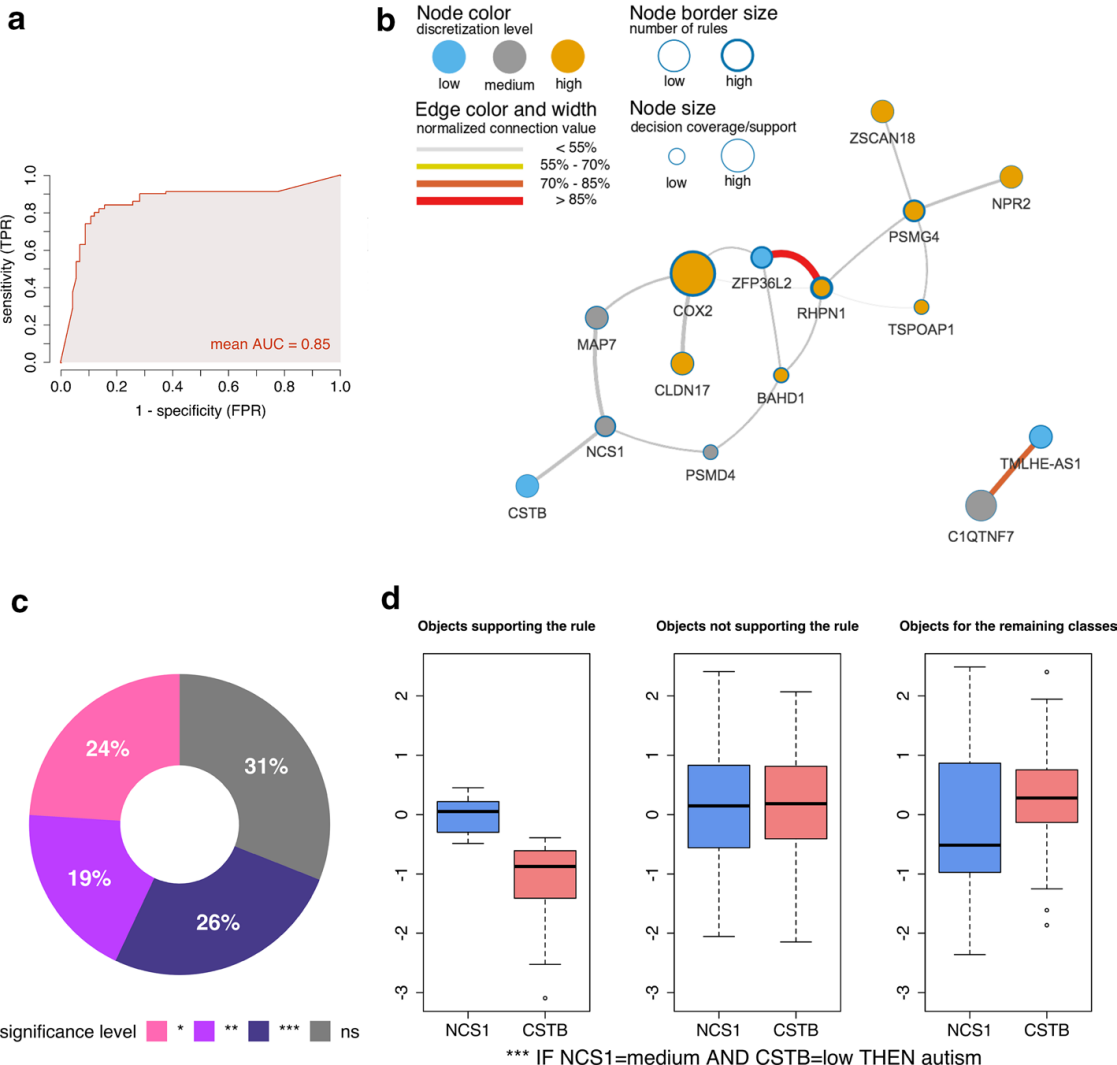
Rule-based model evaluation for the autism-control dataset performed with the Johnson reduction method. Discretization levels were obtained from the equal frequency method by categorizing the features into three bins. **a** ROC AUC for the model. Sensitivity that is as a true positive rate (TPR) and 1-specificity that is a false positive rate (FPR). **b** VisuNet network of co-predictive features for the autism class. Connection values represent the strength of node or edge. These values were estimated based on the rule support and accuracy. Rules were selected based on their statistical significance (Bonferroni-adjusted *P* ≤ 0.05). **c** Distribution of the significance of rules in the model. Bonferroni-adjusted *P* values were marked as ns(*P* > 0.05), *(*P* ≤ 0.05), **(*P* ≤ 0.01) and ***(*P* ≤ 0.001). **d** Distribution of support sets for the top-ranked rule from the recalculated model. Support sets represent sets of objects that fulfil the RHS of the rule (THEN-part). Boxplots display scaled gene expression values for objects supporting and non-supporting the given rule

We constructed two models (Additional file 1: Classification) with R.ROSETTA for Johnson and Genetic reducers with 80% and 90% (0.88 and 0.99 area under the ROC curve) accuracy, respectively. Model significance was determined using a permutation test. The labels for the decision class were randomly shuffled 100 times and a new model was constructed on each modified dataset. We compared these shuffled models to the original (non-shuffled) models, and found that none of the random models resulted in a better accuracy or AUC (*P* ≤ 0.01) (Additional file 1: Fig. S5). To test the influence of undersampling, we generated balanced models with 82% and 90% accuracy (0.85 and 0.98 area under the ROC curve), respectively (Fig. 3a, Additional file 1: Fig. S1a, Table S1). As the difference between unbalanced and balanced data performance was small, we analyzed these models interchangeably. For simplicity and regarding the computation time complexity, undersampling was turned off in models used for permutation tests (Additional file 1: Fig. S5) and benchmarking (Fig. 2, Additional file 1: Fig. S3).

The overall performance of the Genetic algorithm was better than Johnson’s. However, its tendency to generate numerous rules reduced the significance of individual rules after correcting for multiple testing (Fig. 3c, Additional file 1: Fig. S6, Table S1). To identify the most relevant co-predictors among genes, we selected several significant (Bonferroni-adjusted *P* ≤ 0.05) top rules (Fig. 3d, Table 3) from the Johnson model. In addition, for the same model, we presented a sample set of strongly significant (Bonferroni-adjusted *P* < 0.001) rules in Additional file 1: Table S8. The highest ranked co-predictors include the medium expression levels of neuronal calcium sensor 1 (*NCS1*) and low expression levels of cystatin B (*CSTB*). The *NCS1* gene is related to the calcium homeostasis control [65] and is predominantly expressed in neurons [66]. In previous studies, dysregulated expression and mutations in *NCS1* have been linked to neuropsychiatric disorders [65, 66]. Moreover, another study has demonstrated that calcium homeostasis is altered in autism disorders [67]. *CSTB* is a second component of the rule and its elevated expression have been linked to immune response [68]. Furthermore, the reduced expression of *CSTB* has been linked to the mechanism of pathogenesis in epilepsy [69].

We also utilized the VisuNet framework that supports visualization and exploration of rule-based models. Moreover, we displayed a pruned rule-based network for the significant rules for autism (Bonferroni-adjusted *P* ≤ 0.05) (Fig. 3b). The largest node in the network is the cyclooxygenase 2 (*COX2*) gene and suggests a meaningful contribution to the prediction of young males with autism. Elevated expression of *COX2* has been earlier associated with autism [70]. The study reported that *COX2* carried the Single Nucleotide Polymorphism (SNP) rs2745557 and the GAAA haplotype that were significantly associated with autism [70]. Moreover, *COX2* is constitutively expressed in neuronal tissues of patients with psychiatric disorders [71]. Other studies have shown that *COX2*-deficient mice show abnormal expression of autism-related genes [72] and presented its possible therapeutic character for neuropsychiatric disorders [73, 74]. Based on the network, we can also observe a very strong co-prediction between the high expression levels of rhophilin rho GTPase binding protein 1 (*RHPN1*) and the low expression levels of *ZFP36* ring finger protein like 2 (*ZFP36L2*). The association of abnormalities in the GTPase signaling pathway and neurodevelopmental disorders has been previously reported [75]. Rho GTPases participate in the spectrum of signaling pathways related to neurodevelopment such as neurite extension or axon growth and regeneration [75]. The second component is a zinc-finger protein coding gene [76]. The enrichment of lowly expressed zinc fingers in the case–control studies of autism was also discovered by the authors of this dataset [57]. We investigated other autism-related genes that have been reported and described in Additional file 1: Feature validation. The described co-prediction mechanisms illustrate dependencies among the genes that may suggest biological interactions. Although we found relationships to neurodevelopmental and autistic pathways, given hypotheses shall be further verified experimentally.

### Synthetic data evaluation

To explore the influence of the basic properties of the decision table onto the rule-based modelling, we implemented a function that generates synthetic decision tables. We used such synthetic data to describe the rule-based model performance with respect to the number of features, the number of objects and the decision-class imbalance (Additional file 1: Figs. S7, S8). Multiplying the number of features did not affect the quality of the model that remained stable across tests (Additional file 1: Fig. S7b, c). However, increasing the number of objects moderately improved the overall quality of the model (Additional file 1: Fig. S7e, f). To show that undersampling corrects biased performance that arose from the class imbalance, we generated random synthetic datasets with various imbalance proportions. We showed that the class imbalance issue biases the accuracy and the bias is corrected after applying the undersampling (Additional file 1: Fig. S8). We also confirmed that it is better to assess the performance with the AUC values, which are immune to uneven distribution of samples (Additional file 1: Fig. S8).

## Conclusions

The R.ROSETTA is a response to the needs of developers of interpretable machine learning models for Life Sciences. It facilitates the access to the functionality of the R environment that is one of the major environments used in bioinformatics. To our knowledge, it is the first and only learning system that makes available a comprehensive toolbox of statistical measures essential in analyzing, validating and, potentially, certifying classifiers at the level of the models and their components. Furthermore, several original ROSETTA procedures were improved and/or adapted to the R environment and target bioinformatics applications. These improvements include undersampling methods to account for imbalance, estimation of the statistical significance of classification rules, retrieving objects from support sets, normalized prediction of external datasets and integration with rule-based visualization tools.

Rule-based models generated under the paradigm of rough sets have several attractive properties but also limitations. Models are built using a well-defined procedure of Boolean reasoning to obtain reducts i.e. minimal subsets of the original set of features that maintain discernibility of the decision classes; usually, approximate reducts are generated, both for the sake of computational efficiency but also for their often better generalization properties. Rough sets are especially useful in the applications to modelling biological systems since living organisms are robust and often have multiple ways of achieving their goals. These may be captured by multiple reducts. The price for finding all possible reducts is the complexity of the problem, which is NP-hard**,** while finding only one minimal subset of features (corresponding to finding one reduct in R.ROSETTA) is linear in time. Another disadvantage of R.ROSETTA may be the very large number of rules generated with the Genetic heuristics which makes such models more difficult to interpret. However, we showed that with the use of the toolbox it is possible to prune such model**s** and keep the statistically significant rules. Another feature of the rough set-based models is the need to discretize the values of the features. Depending on the application this at times may hamper the quality of classification, but equally well, this may improve interpretation of the models and their generalization power.

Herein, we investigated the package to describe the influence of the properties of decision tables on the rule-based learning performance. Next, a real sample application of analyzing autism and controls using gene expression data was introduced and the classifier interpreted. The autism-control dataset was also exploited to benchmark the package with a broad selection of the state-of-the-art methods within the rule- and decision tree-based domains. R.ROSETTA compared favorably with the other methods with the Genetic heuristics usually outperforming the Johnson heuristics; both heuristics compared favorably with the other systems. We showed that a rule set, be it generated with any of the two heuristics, can be visualized in the form of co-predictive rule-networks, which further enhance the interpretability of rule-based models. Finally, we investigated the performance of R.ROSETTA depending on the properties of the decision tables that are input to the system.

In contrast to methods that allow explaining black box approaches, so-called post hoc explanation methods, rough sets theory is a technique that directly produces interpretable models. On the other hand, commonly used algorithms, such as ELI5 [77], LIME [78] or SHAP [79], are able to explain most of machine learning models. However, recent studies have shown that explanations of black box models may be affected by biases and their application has been questioned [80, 81]. Nevertheless, interpretable models and explainable methods have a common goal of elucidating classifications and are likely to complement each other.

## Supplementary Information

**Additional file 1.** Supplementary Notes, Supplementary Figures S1–S8, Supplementary Tables S1–S8, Supplementary References

## Data Availability

The data that support the findings of this study are publicly available from the Gene Expression Omnibus repository, [GSE25507, https://www.ncbi.nlm.nih.gov/geo/query/acc.cgi?acc=GSE25507]. The synthetic data that supports the findings of this study has been generated with the R.ROSETTA package. The package can be found at https://github.com/komorowskilab/R.ROSETTA.

## Abbreviations

AUC: Area under the ROC curve
AQ: AQ method
C50: C.50 method
CN2: Clark and Niblett method
CV: Cross validation
FPR: False positive rate
GBM: Generalized boosted models
JRip: Repeated Incremental Pruning to Produce Error Reduction—RIPPER
Lem2: Learning from Examples Module—version 2
LHS: Left-hand site
ns: Not significant
OneR: 1R classifier method
PART: Partial decision trees-based method
*P*: *P* Value
RHS: Right-hand site
ROC: Receiver Operating Characteristic
TPR: True positive rate
