# Supplementary material for: R.ROSETTA: an interpretable machine learning framework
Source: BMC Bioinformatics. 2021 Mar 6;22:110. doi: 10.1186/s12859-021-04049-z (PMC7937228; doi:10.1186/s12859-021-04049-z)
Supplement: Supplementary file 1 — Additional file 1. Supplementary Notes, Supplementary Figures S1–S8, Supplementary Tables S1–S8, Supplementary References [file 12859_2021_4049_MOESM1_ESM.pdf]

Additional file 1

Supplementary material

R.ROSETTA: an interpretable machine learning framework

Mateusz Garbulowski[1], Klev Diamanti[1,2,#], Karolina Smolińska[1,#], Nicholas Baltzer[1,3], Patricia Stoll[1,4], Susanne Bornelöv[1,5], Aleksander Øhrn[6], Lars Feuk[2] and Jan Komorowski[1,7,8,9,*]

[1]Department of Cell and Molecular Biology, Uppsala University, Sweden, [2]Department of Immunology, Genetics and Pathology, Uppsala University, Sweden, [3]Department of Research, Cancer Registry of Norway, Norway, [4]Department of Biosystems Science and Engineering, ETH Zurich, Switzerland, [5]Present address: Cancer Research UK Cambridge Institute, University of Cambridge, UK, [6]Department of Informatics, University of Oslo, Norway, [7]Swedish Collegium for Advanced Study, Uppsala, Sweden, [8]Institute of Computer Science, Polish Academy of Sciences, Poland, [9]Washington National Primate Research Center, Seattle, WA, USA

*Correspondence: jan.komorowski@icm.uu.se (Jan Komorowski)

[#]These authors contributed equally to the work

R.ROSETTA is freely available at https://github.com/komorowskilab/R.ROSETTA

Tutorials and more information can be found at https://komorowskilab.github.io/R.ROSETTA/

Supplementary notes 2

- Package architecture 2
- Benchmarking 2
- Data preprocessing 2
- Feature selection 2
- Classification 3
- Feature validation 3

Supplementary figures 4

Supplementary tables 12

Supplementary references 20

Supplementary notes

Package architecture

The ROSETTA framework comes in a GUI version for Windows systems and a command line version for UNIX-based systems. The R.ROSETTA package is a cross-platform application that uses command line ROSETTA. However, UNIX-based systems require installation of the compatibility layer software Wine (https://www.winehq.org/). For more information we recommend to read the original ROSETTA articles [1-3] and the technical reference manual [4]. Detailed instructions for the R.ROSETTA installation, functions and a sample code are available in the package manual or on the official R.ROSETTA website (https://komorowskilab.github.io/R.ROSETTA/).

Benchmarking

We compared R.ROSETTA with three other R packages designed for rule-based modelling. Herein, we excluded algorithms that focus on fuzzy rule-based learning, operate on continuous decision classes and/or include fixed internal discretization methods. In addition, we compared R.ROSETTA with four popular decision tree-based methods. The parameters of each methods were either set to default or tuned for more exhaustive search. We standardized the learning procedure for all methods so that each was performed with 10-fold CV and equal frequency discretization. In the discretization part, cuts were estimated based on a training set and further applied to discretize the test set. Each method was repeated 20 times with a different seed level. The runtime of each method was measured as a time between inputting a data to the function and obtaining a rule-based or tree-based model. Number of rules and runtime were calculated as an average value from 20 repetitions. The calculations were performed with macOS High Sierra with the following parameters: processor 2,2 GHz Intel Core i7 and memory 8GB 1600MHz DDR3. All the benchmarking methods were run with autism-control data that contains preselected set of features.

Data preprocessing

The so-called autism-control dataset was loaded and processed using the getGEO function from the GEOquery R library [5]. The data was normalized with the Robust Multi-array Average (RMA) functions *ReadAffy* and *rma* from the affy R package [6]. Gene names were retrieved using AnnotationDbi [7] and hgu133plus2.db [8] R packages. To identify unknown probe names, the annotation table HG-U133_Plus_2.na36 downloaded from http://www.affymetrix.com/site/mainPage.affx was processed and the probe coordinates were intersected with the unknown probes using the GenomicRanges [9] R package. For one probe the gene name could not be identified due to the lack of coordinates. The clinical data was investigated for potential batch effects using the Pearson correlation with the rcorr function from the Hmisc R library [10]. The age of the samples was highly correlated to the outcome. The sva R package [11] was used to correct the data for the age effect.

Feature selection

Dimensionality reduction of the autism-control dataset was performed with the Fast Correlation-Based Filter (FCBF) method [12] available as a function in the Biocomb R package [13]. The feature selection method utilized the predominant correlation of the features and decision along with the redundancy. We chose FCBF as the method that selected the highest number of important features among the tested software. The other advantage of using FCBF was the compatibility of the discretization method with R.ROSETTA. The FCBF function was set to Equal Frequency discretization for three levels. The method selected 35 features with Information Gain above 0. This step could be alternatively performed with other dimensionality

reduction methods such as Monte Carlo Feature Selection [14, 15], Boruta [16], Student's t-test, caret R package [17] and many others.

Classification

The final decision table was constructed using the most informative 35 genes, 146 objects/samples and the decision class (autism or control). The models were created using 10-fold cross validation (CV) for the standard voter method, equal frequency discretization into three levels, discernibility of the objects option and Bonferroni method to adjust rule *P* values for multiple testing. We choose to use Bonferroni correction in order to rigorously adjust large numbers of generated rules, especially in case of Genetic algorithm. However, type of *P* value correction can be easily changed in the R.ROSETTA parameters. Moreover, in some cases, undersampling was applied to remove a slight imbalance between the proportions of decision classes. Herein, the paper focuses on Johnson and Genetic reduction methods. However, for simplicity of the model and rule significance reasons most of the interpretations were based on the rules estimated from the Johnson reducer.

Feature validation

We described sample genes that were likely associated with autism in the results section of the main article. Additionally, we depicted genes that had been earlier linked to the brain or the nervous system such as: migraine, headache – *PPOX* [18], smell perception – *OR51B5* [19] and ataxia – *ATXN8OS* [20]. We found that *NCKAP5L* is a gene likely to be involved in neurodevelopmental dysfunction in autism [21]. We showed that expression of *NCKAP5L* gene was down-regulated in non-autistic patients. Among the most relevant features, we discovered a group of zinc fingers [22] such as *ZSCAN18*/*ZNF447*, *ZFP36L2*, and *KLF8*/*ZNF741* and a group of genes related to calcium homeostasis control [23] such as *SCIN*, *NCS1*, and *CAPS2*. Moreover, we found co-prediction mechanisms among the genes that were not previously reported as autism-related genes e.g. proteasome assembly chaperone 4 (*PSMG4*) or bromo adjacent homology domain containing 1 (*BAHD1*) (see Fig. 3b).

Supplementary figures

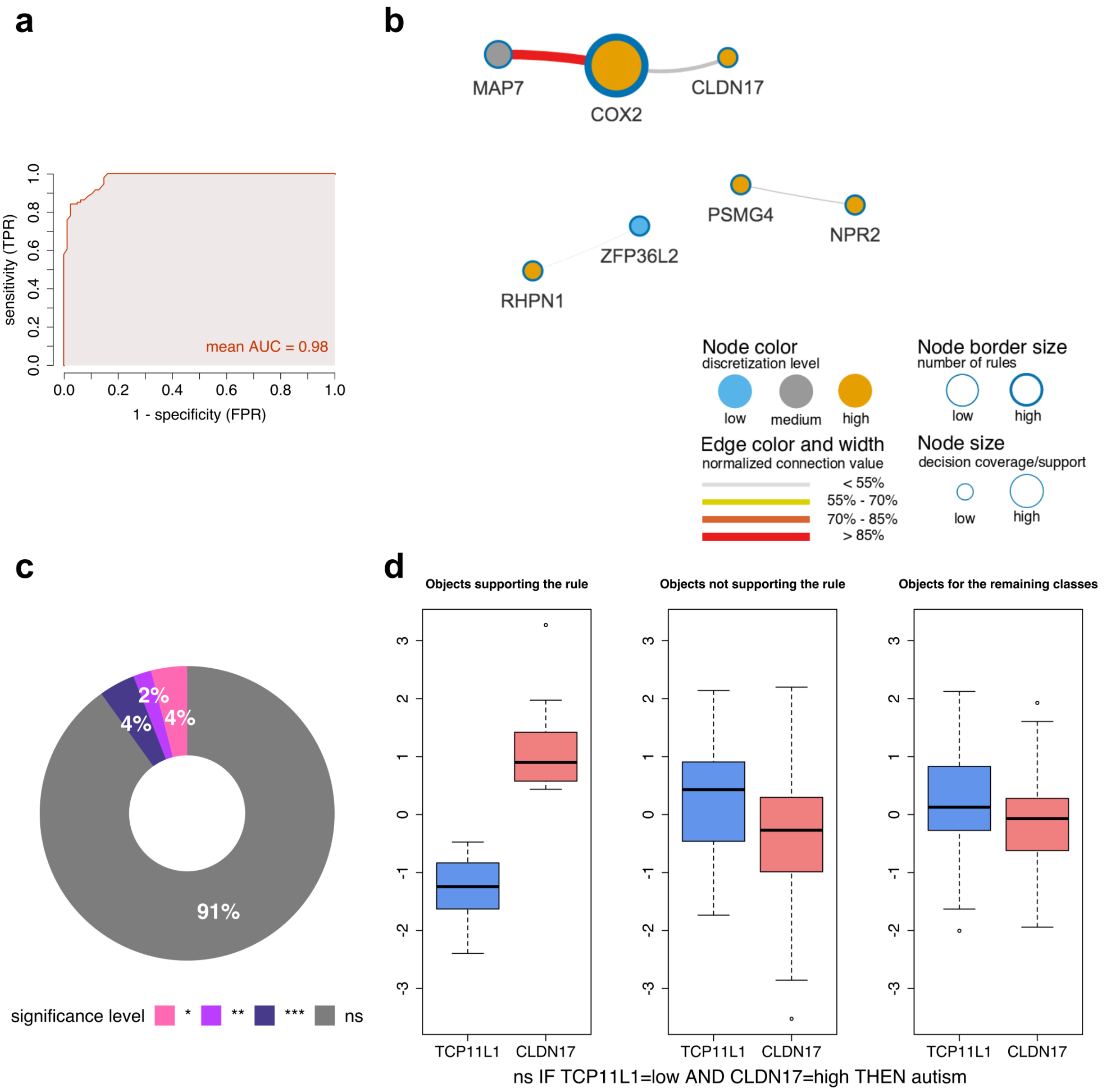

Fig. S1. Rule-based model evaluation for the autism-control dataset performed with with the Genetic reduction method. Discretization levels were obtained from the equal frequency method by categorizing the features into three bins. **a** Area under the ROC curve (AUC) for the model. Sensitivity that is as a true positive rate (TPR) and 1-specificity that is a false positive rate (FPR). **b** VisuNet network of co-predictive features for the autism class. Connection values represent the strength of node or edge. These values were estimated based on the rule support and accuracy. Rules were selected based on their statistical significance (Bonferroni-adjusted $P \leq 0.05$). **c** Distribution of the significance of rules in the model. Bonferroni-adjusted P values were marked as ns($P > 0.05$), *($P \leq 0.05$), **($P \leq 0.01$) and ***($P \leq 0.001$). **d** Distribution of support sets for the top-ranked rule from the recalculated model. Support sets represent sets of objects that fulfil the RHS of the rule (THEN-part). Boxplots display scaled gene expression values for objects supporting and non-supporting the given rule.

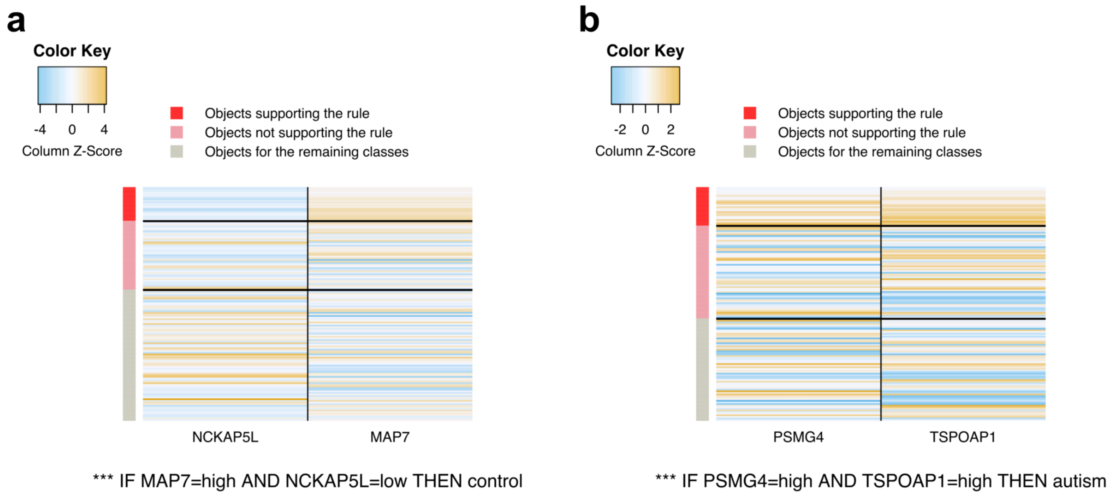

Fig. S2. A rule-oriented graphic representation of its corresponding continuous values from the decision table. A given rule comes from the recalculated autism-control model. **a** The most significant co-predictors for the control class. **b** The most significant co-predictors for the autism class

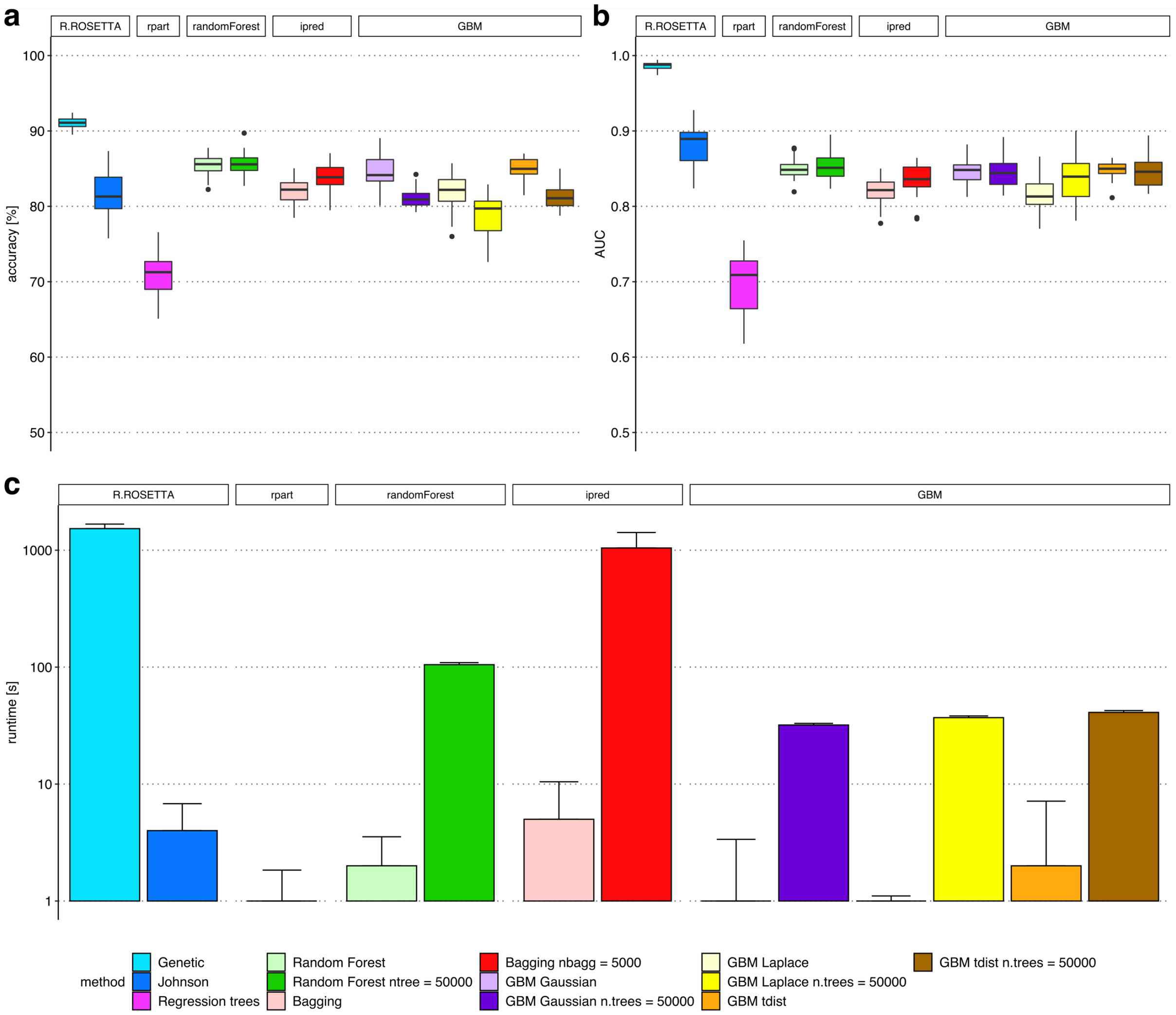

Fig. S3. Benchmarking the autism-control dataset with the R packages for decision trees methods. The packages were evaluated with regression trees, random forest, bagging and generalized boosted regression models (GBM). Several methods were tuned for number of bootstrap replications (*nbagg*) and number of trees (*ntree* and *n.trees*). Other methods were evaluated with default parameters. The results of benchmarking are presented for **a** accuracy distribution of classifiers, **b** area under the ROC curve (AUCs) distribution of classifiers, **c** number of estimated rules (logarithmic scale) and **d** average runtime of the algorithms (logarithmic scale). Two standard deviations were marked above each bar. The time was measured from inputting a decision table to receiving a model.

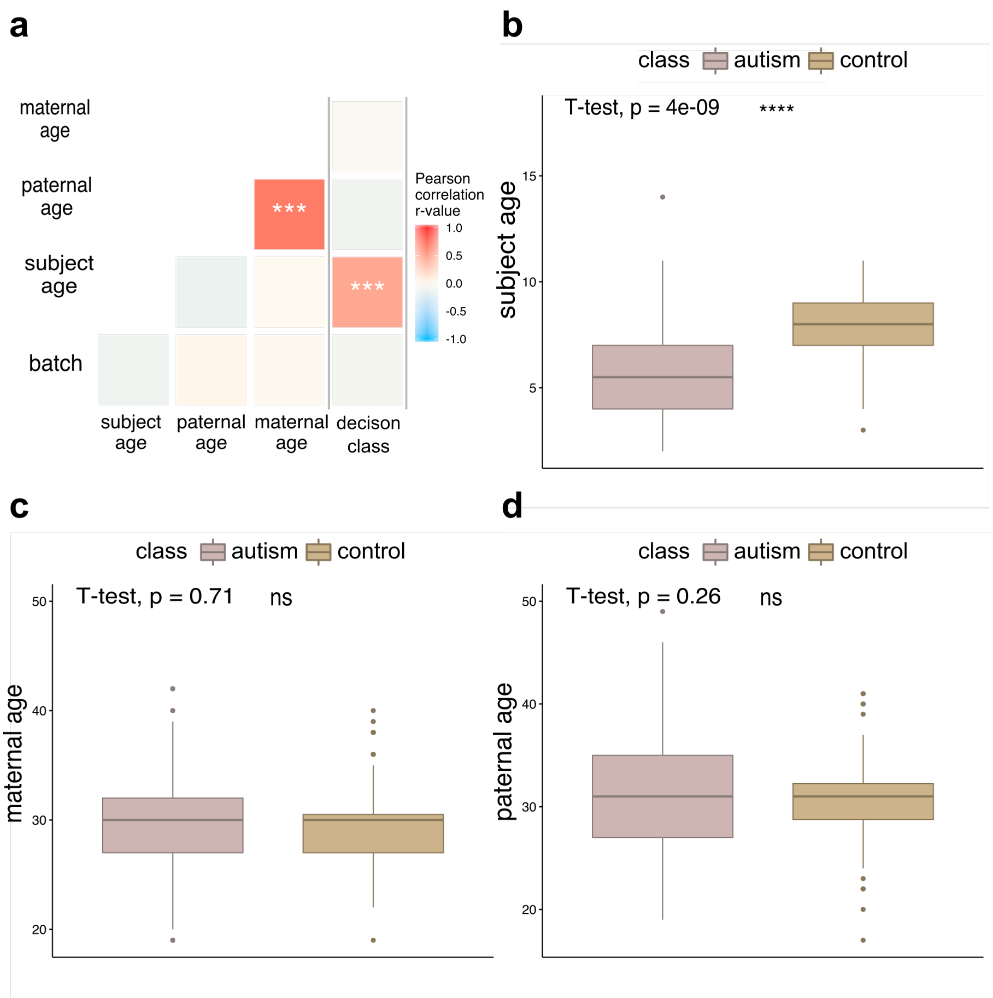

Fig. S4. a Pearson correlation r values derived from the clinical data. Stars represent the *P* value significance level of the correlation values, denoted by non-significant: ns($P > 0.05$) and significant: *($P \leq 0.05$), **($P \leq 0.01$), ***($P \leq 0.001$). The decision column is detached with dark blue lines to illustrate the effect of the clinical data on the decision. **b** Relationship between outcome and subject age (in years) **c** Relationship between the outcome and maternal age (in years). **d** A relationship between outcome and paternal age (in years). The Student's t-test was used to test for a difference in subject, maternal or paternal age between cases and controls in **b**, **c** and **d** with *P* values indicated in the figure.

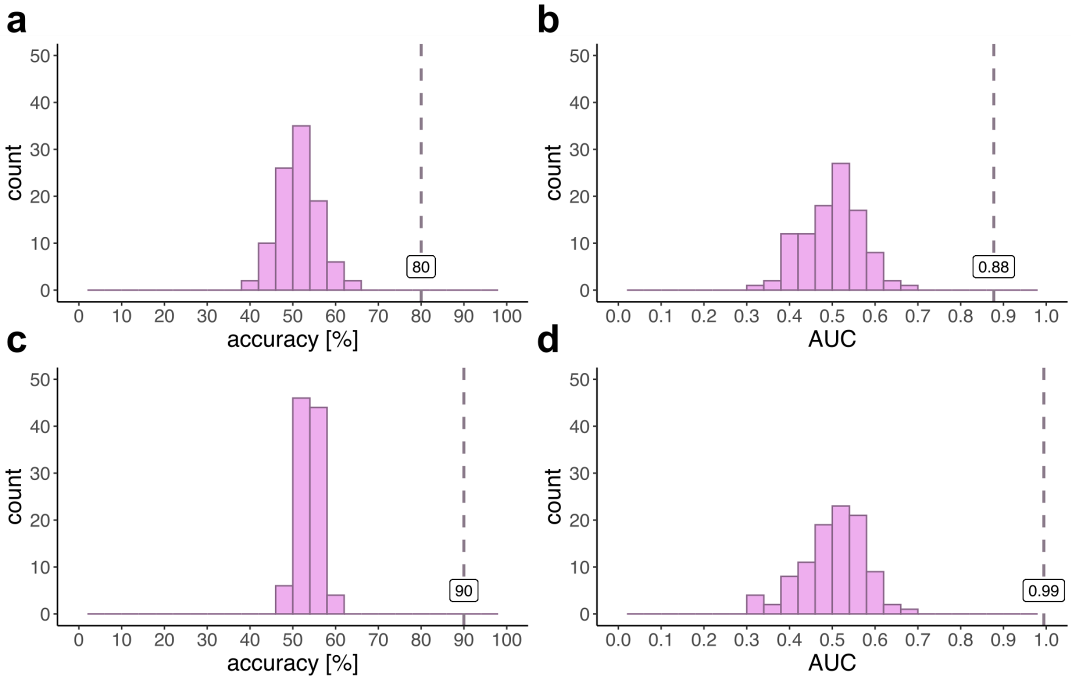

Fig. S5. Permutation tests for rule-based models of the autism-control performed with the (**a, b**) Johnson and (**c, d**) Genetic reduction method. Each histogram represents 100 permutations. Dashed line indicates quality measures of non-permuted models. Accuracy and area under the ROC curve (AUC) were used to compare the performance of classifiers.

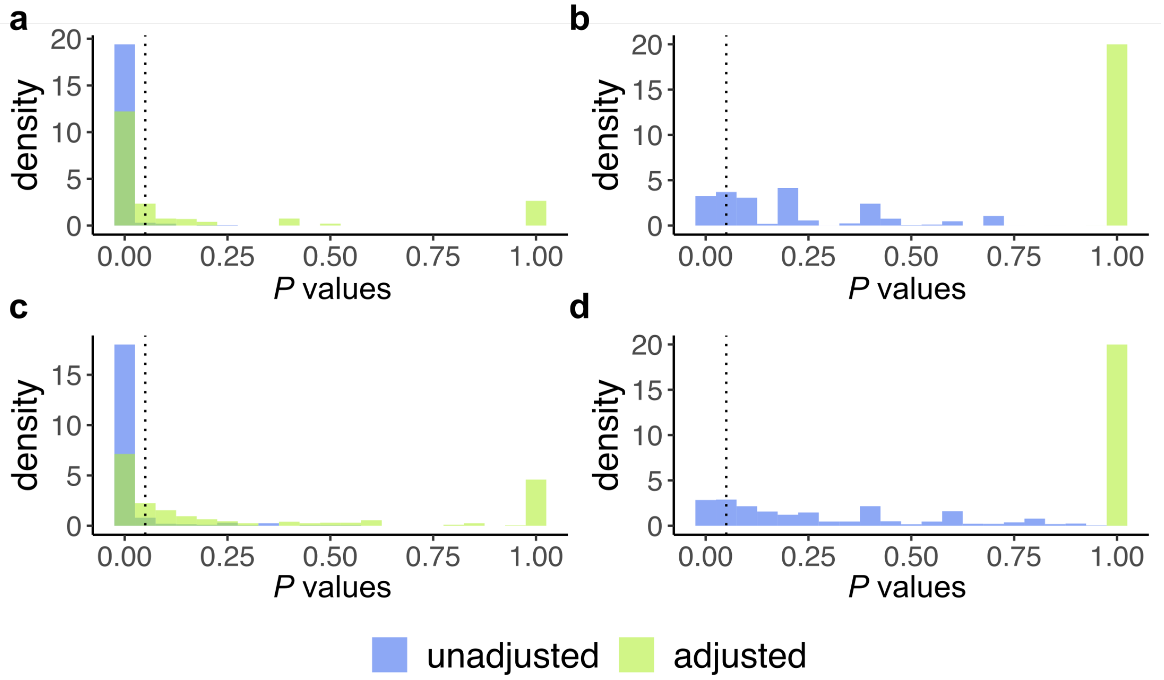

Fig. S6. The density of rule *P* values for the reduction methods. Histograms display the comparison of the *P* value adjustment and the model recalculation between reducers. A dotted line marks the 0.05 significance threshold. **a** Autism-control basic model generated with the Johnson reducer method. **b** Autism-control basic model generated with the Genetic reducer method. **c** autism-control recalculated model with the Johnson reducer method. **d** Autism-control recalculated model with the Genetic reducer method.

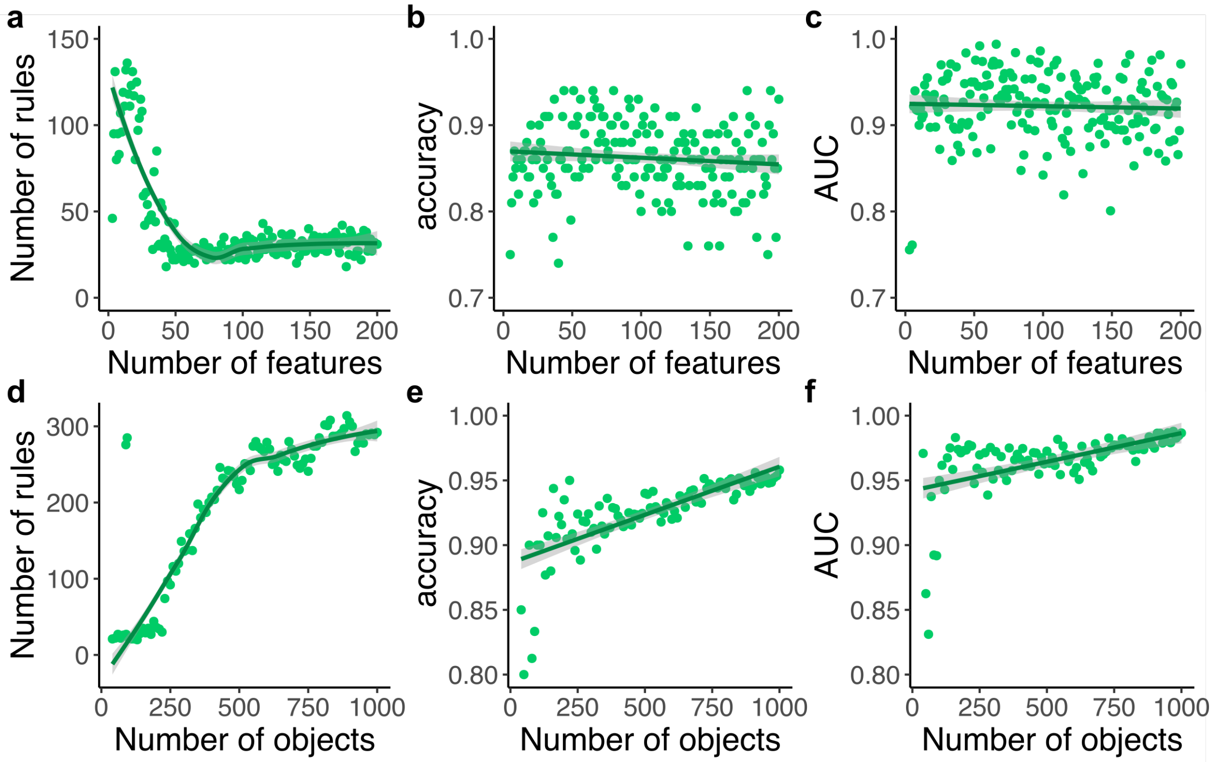

Fig. S7. The impact of number of features (**a-c)** and objects (**d-f)** on the rule-based model quality. The tests were performed with the Johnson reduction algorithm on the synthetic data with a feature-feature correlation, $r_f = 0.4$ and feature-decision correlation, $r_d = 0.6$.

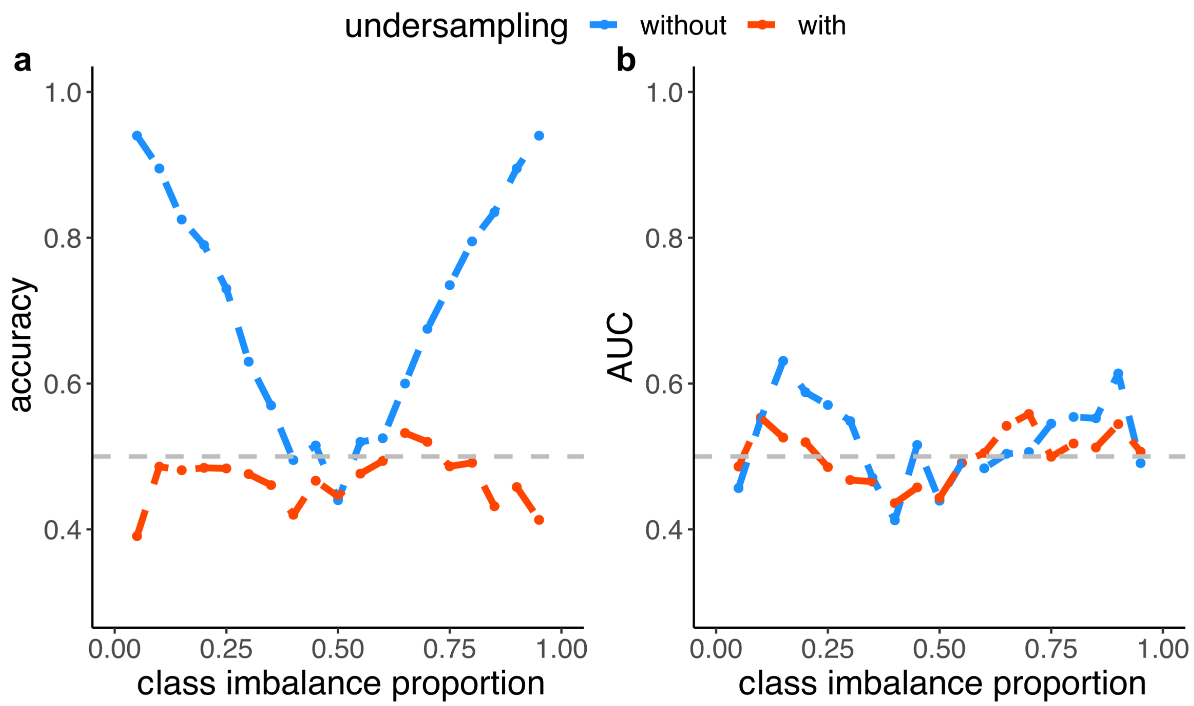

Fig. S8. An undersampling application for various class imbalance proportions. The tests were performed with the Johnson reduction algorithm on the synthetic data with a random correlation to the outome (expected accuracy and AUC are 0.5). The synthetic data consisted of 50 features and 100 objects. The class imbalance proportion was established from 0.05 to 0.95 with a 0.05 step. **a** The relation between the class imbalance with/without undersampling and model accuracy **b** The relation between the class imbalance with/without undersampling and the area under the ROC curve (AUC).

Supplementary tables

Table S1. A comparison of the efficiency of the Johnson and Genetic reducers for basic (not recalculated) and recalculated rules. The data was discretized using the Equal Frequency method and the model was constructed with 10-fold CV of the standard voter classification method. The model was balanced using undersampling due to slightly imbalanced distribution of classes. The obtained *P* values were Bonferroni-adjusted.

**reducer type**	Johnson	Genetic
**mean accuracy**	82%	90%
**mean AUC**	0.85	0.98
**the total number of rules**	401	156650
	basic	
**number of rules ns($P > 0.05$)**	123	156645
**number of rules *($P \leq 0.05$)**	278	5
**number of rules **($P \leq 0.01$)**	182	3
**number of rules ***($P \leq 0.001$)**	104	2
	recalculated	
**number of rules ns($P > 0.05$)**	218	156641
**number of rules *($P \leq 0.05$)**	183	9
**number of rules **($P \leq 0.01$)**	111	1
**number of rules ***($P \leq 0.001$)**	39	0

Table S2. Performance evaluation of rules for the Genetic reduction method with undersampling. The average statistic values of rule support and accuracy are presented in the table. For the rule statistics, the most significant co-predictors (Bonferroni-adjusted $P \leq 0.05$) were selected.

**class**	control		autism	
**total number of rules**	75530		81120	
**rule statistics**	basic	recalculated	basic	recalculated
**number of rules (*P* ≤ 0.05)**	2	8	3	1
**LHS support**	4	6	5	6
**RHS support**	4	5	4	4
**accuracy**	0.81	0.80	0.86	0.74
**top co-predictors**	MAP7, NCKAP5L	MAP7, NCKAP5L	MAP7, COX2	TCP11L1,CLDN17 RHPN1,PPOX

Table S3. The performance evaluation of vote normalization methods in reclassifying the autism-control dataset. Bonferroni-adjusted P value ≤ 0.05 based filtration was investigated for the Johnson and Genetic reducers. The vote counts were normalized by different factors: median, mean, maximum (max), square root of the sum of squares (srss) or rule number (rulnum). The values represent accuracy (ACC) and AUC measures.

**method**	none				median				mean				max				srss				rulnum			
**rules**	basic		recalculated		basic		recalculated		basic		recalculated		basic		recalculated		basic		recalculated		basic		recalculated	
**quality**	ACC	AUC	ACC	AUC	ACC	AUC	ACC	AUC	ACC	AUC	ACC	AUC	ACC	AUC	ACC	AUC	ACC	AUC	ACC	AUC	ACC	AUC	ACC	AUC
**Johnson**	95%	0.95	96%	0.96	95%	0.95	92%	0.93	96%	0.96	96%	0.96	96%	0.96	96%	0.96	96%	0.96	96%	0.96	90%	0.91	96%	0.95
**Genetic**	71%	0.73	49%	0.55	71%	0.71	67%	0.70	72%	0.73	67%	0.70	72%	0.73	67%	0.70	72%	0.73	67%	0.70	70%	0.70	49%	0.55

Table S4. A comparison of the R.ROSETTA package to other R packages that enable rule-based classification modelling. The average accuracy, AUC, number of rules and time was calculated from the models with 20 repetitions of 10-fold CV without undersampling. The tests were performed on the autism-control dataset. The time was measured with tictoc library [24] as a time required for building a model to calculate the model quality measures.

**R package**	C50	RoughSets			R.ROSETTA		RWeka		
**package author**	*Kuhn, M. et al. (2018)*	*Riza, L. S. et al. (2014)*			*Garbulowski, M. et al. (2021)*		*Hornik, K., Buchta, C. and Zeileis, A. (2009)*		
**algorithm abbreviation**	C50	AQ	CN2	LEM2	GenR	JohnR	JRip	OneR	PART
**detailed name of the algorithm**	C5.0	quasi-optimal covering	CN2 rule induction	Learning from Examples Module – version 2	Genetic reducer	Johnson reducer	Repeated Incremental Pruning to Produce Error Reduction – RIPPER	1R classifier	partial decision trees
**algorithm author**	*Quinlan, J.R. (1992)*	*Michalski, R.S. et al. (1991)*	*Clark, P.E. and Niblett, T. (1989)*	*Grzymala-Busse J.W. (1997)*	*Wroblewski, J. (1995)*	*Johnson, D.S. (1974)*	*Cohen, W.W. (1995)*	*Holte, R.C. (1993)*	*Frank, E. and Witten, I.H. (1998)*
**function name**	C5.0.default	AQRules.RST	RI.CN2Rules.RST	RI.LEM2Rules.RST	rosetta		Jrip	OneR	PART
**discretization**	equal frequency								

Table S5. A correlation between the outcome and clinical data of the autism-control dataset. The *P* values were estimated with the Student's t-test. Non-significant differences are shown between batches and parental ages. The highly significant correlation for the age of subjects and outcome is shown.

**decision class**	control	autism	*P* value
**number of samples**	64	82	-
**subject age**	7.9±2.1	5.5±2.1	4.26×10^{-9}
**maternal age**	29.7±5	30.1±5.6	0.71
**paternal age**	30.3±5.3	31.5±6.3	0.27
**batch**	B1(32), B2(32)	B1(36), B2(46)	0.46

Table S6. A comparison of the performance of four dimensionality reduction methods applied to the autism-control dataset. For Boruta and Student's t-test the P value ≤ 0.05 threshold was established. The FCBF method selected genes with the Information Gain (IG) greater than 0. Monte Carlo Feature Selection (MCFS) estimated a Relative Importance (RI) threshold with a critical angle method.

**feature selection method**	Boruta	FCBF	MCFS	Student's t-test
**R package**	Boruta	Biocomb	rmcfs	stats
**threshold**	$P < 0.05$	IG > 0	RI > 0.036	$P < 0.05$
**number of features**	12	35	16	13
**discretization**	No	Yes	No	No

Table S7. The result of the FCBF method applied on the autism-control dataset. The list is decreasingly sorted from the features with the highest Information Gain (IG). The position of each feature in a ranking is given in the first column. The translation between microarray probe ID and gene ID is shown in the last two columns.

position	IG	gene ID	probe ID
1	1.09e-01	MAP7	202890_at
2	9.95e-02	COX2	1553569_at
3	9.45e-02	NCKAP5L	1562457_at
4	8.57e-02	ZSCAN18	217593_at
5	8.39e-02	RHPN1	235998_at
6	7.77e-02	PPOX	238118_s_at
7	7.63e-02	NPR2	204310_s_at
8	7.61e-02	NCS1	222570_at
9	7.13e-02	PSMG4	233443_at
10	6.79e-02	SCIN	1552367_a_at
11	6.56e-02	CSTB	236449_at
12	6.24e-02	TSPOAP1	205839_s_at
13	6.22e-02	TCP11L1	205796_at
14	6.19e-02	234817_at	234817_at
15	6.12e-02	TMLHE-AS1	1560797_s_at
16	6.06e-02	PSMD4	200882_s_at
17	5.98e-02	ZFP36L2	201367_s_at
18	5.94e-02	B3GNT7	1555962_at
19	5.75e-02	MSI2	225238_at
20	5.73e-02	CAPS2	224370_s_at
21	5.70e-02	MIR646HG	1562051_at
22	5.58e-02	CLDN17	221328_at
23	5.51e-02	BAHD1	203051_at
24	5.29e-02	OR51B5	1570516_s_at
25	5.08e-02	C11orf95	218641_at
26	4.91e-02	ATXN8OS	216404_at
27	4.74e-02	NRG2	242303_at
28	4.69e-02	LOC400655	216703_at
29	4.69e-02	GJA9	221415_s_at
30	4.47e-02	VPS8	234028_at
31	4.34e-02	FLRT2	240259_at
32	3.89e-02	C1QTNF7	239349_at
33	3.61e-02	KLF8	219930_at
34	3.57e-02	CWF19L2	1566515_at
35	3.12e-02	DEPDC1	222958_s_at

Table S8. Set of rules and their statistics from autism-control model estimated with Johnson reducer. Rules were selected from undersampled and recalculated model with Bonferroni-adjusted *P* value < 0.001. Discretization bins were estimated with equal frequency method and represent low, medium and high gene expression.

no	rule	length	accuracy	RHS support	*P* value
1	IF MAP7=high AND NCKAP5L=low THEN control	2	0,95	21	1.99e-05
2	IF ZSCAN18=low AND NPR2=medium THEN control	2	1	18	3.03e-05
3	IF PPOX=low AND OR51B5=medium THEN control	2	1	18	3.03e-05
4	IF NCS1=medium AND CSTB=low THEN autism	2	1	25	3.24e-05
5	IF NCKAP5L=low AND NCS1=low THEN control	2	0,92	22	5.84e-05
6	IF COX2=high THEN autism	1	0,87	40	6.07e-05
7	IF PSMG4=high AND TSPOAP1=high THEN autism	2	1	24	6.82e-05
8	IF NPR2=medium AND CAPS2=high THEN control	2	1	17	8.33e-05
9	IF MAP7=high AND ATXN8OS=low THEN control	2	1	17	8.33e-05
10	IF NCKAP5L=low AND MSI2=high THEN control	2	1	17	8.33e-05
11	IF NCKAP5L=low AND B3GNT7=low THEN control	2	1	17	8.33e-05
12	IF PPOX=low AND NCS1=low THEN control	2	0,88	23	1.28e-04
13	IF ZSCAN18=low AND C11orf95=low THEN control	2	0,95	19	1.45e-04
14	IF MAP7=high AND PPOX=low THEN control	2	0,91	21	1.54e-04
15	IF NCKAP5L=low AND PPOX=low THEN control	2	0,91	21	1.54e-04
16	IF NPR2=medium AND NCS1=low THEN control	2	0,91	21	1.54e-04
17	IF PSMG4=medium AND MSI2=high THEN control	2	1	16	2.26e-04
18	IF NCKAP5L=low AND OR51B5=medium THEN control	2	1	16	2.26e-04
19	IF MAP7=medium AND COX2=high THEN autism	2	0,96	26	2.27e-04
20	IF NPR2=medium THEN control	1	0,74	34	2.74e-04
21	IF TCP11L1=low AND CLDN17=high THEN autism	2	1	22	2.94e-04
22	IF RHPN1=high AND PPOX=medium THEN autism	2	1	22	2.94e-04
23	IF PPOX=low AND LOC400655=medium THEN control	2	0,95	18	3.80e-04
24	IF RHPN1=high AND DEPDC1=low THEN autism	2	0,96	25	4.61e-04
25	IF MAP7=medium AND NCS1=medium THEN autism	2	1	21	6.02e-04
26	IF RHPN1=high AND FLRT2=high THEN autism	2	1	21	6.02e-04
27	IF SCIN=medium AND TCP11L1=low THEN autism	2	1	21	6.02e-04
28	IF ZSCAN18=high AND NPR2=high THEN autism	2	1	21	6.02e-04
29	IF COX2=high AND ZFP36L2=low THEN autism	2	1	21	6.02e-04
30	IF NCKAP5L=low THEN control	1	0,72	34	7.05e-04
31	IF PPOX=low AND MSI2=high THEN control	2	0,88	21	8.20e-04
32	IF RHPN1=high AND PSMG4=high THEN autism	2	0,93	27	8.84e-04
33	IF TMLHE-AS1=low AND C1QTNF7=medium THEN autism	2	0,96	24	9.26e-04
34	IF RHPN1=high AND BAHD1=high THEN autism	2	0,96	24	9.26e-04
35	IF COX2=high AND NCS1=medium THEN autism	2	0,96	24	9.26e-04
36	IF 234817_at=low AND TMLHE-AS1=high THEN control	2	0,94	17	9.83e-04
37	IF MAP7=high AND TSPOAP1=medium THEN control	2	0,94	17	9.83e-04
38	IF PPOX=low AND KLF8=medium THEN control	2	0,94	17	9.83e-04
39	IF NCKAP5L=low AND 234817_at=low THEN control	2	0,94	17	9.83e-04

Supplementary references

1. Øhrn A, Komorowski J, Skowron A, Synak P: **The design and implementation of a knowledge discovery toolkit based on rough sets-The ROSETTA system**. In.; 1998.
2. Øhrn A: **The Rosetta C++ Library: Overview of files and classes**. *Department of Computer Information Science, Norwegian University of Science Technology , Trondheim, Norway* 2000.
3. Øhrn A: **Discernibility and rough sets in medicine: tools and application**. *Norwegian University of Science Technology, Norway* 1999:41-51.
4. Øhrn A: **Rosetta technical reference manual**. *Department of Computer Information Science, Norwegian University of Science Technology, Trondheim, Norway* 2001.
5. Davis S, Meltzer PS: **GEOquery: a bridge between the Gene Expression Omnibus (GEO) and BioConductor**. *Bioinformatics* 2007, **23**(14):1846-1847.
6. Gautier L, Cope L, Bolstad BM, Irizarry RA: **affy--analysis of Affymetrix GeneChip data at the probe level**. *Bioinformatics* 2004, **20**(3):307-315.
7. Pages H, Carlson M, Falcon S, Li NA, AnnotationDbi P, SQLForge P: **Annotation database interface**. *R package version* 2008, **1**(2).
8. Carlson M: **hgu95av2. db: Affymetrix Human Genome U95 Set annotation data (chip hgu95av2)**. 2016.
9. Lawrence M, Huber W, Pages H, Aboyoun P, Carlson M, Gentleman R, Morgan MT, Carey VJ: **Software for computing and annotating genomic ranges**. *PLoS computational biology* 2013, **9**(8):e1003118.
10. Harrell FE, Dupont C: **Hmisc: harrell miscellaneous**. In., vol. 3, R package version 4.1-1 edn; 2008.
11. Leek JT, Johnson WE, Parker HS, Jaffe AE, Storey JD: **The sva package for removing batch effects and other unwanted variation in high-throughput experiments**. *Bioinformatics* 2012, **28**(6):882-883.
12. Yu L, Liu H: **Feature selection for high-dimensional data: A fast correlation-based filter solution**. In: *Proceedings of the 20th international conference on machine learning (ICML-03): 2003*. 856-863.
13. Novoselova N, Wang J, Pessler F, Klawonn F: **Biocomb: Feature Selection and Classification with the Embedded Validation Procedures for Biomedical Data Analysis**. In., R Package Version 0.4. edn; 2018.
14. Dramiński M, Kierczak M, Koronacki J, Komorowski J: **Monte Carlo feature selection and interdependency discovery in supervised classification**. In: *Advances in Machine Learning II.* Springer; 2010: 371-385.
15. Dramiński M, Koronacki J: **rmcfs: An R Package for Monte Carlo Feature Selection and Interdependency Discovery**. *Journal of Statistical Software* 2018, **85**(1):1-28.
16. Kursa MB, Rudnicki WR: **Feature selection with the Boruta package**. *J Stat Softw* 2010, **36**(11):1-13.
17. Kuhn M: **Building predictive models in R using the caret package**. *Journal of statistical software* 2008, **28**(5):1-26.
18. Makki AY, Leddy J, Takano K, Jain R: **An Unusual Cause of Headache and Fatigue in a Division 1 Collegiate Athlete**. *Clinical Journal of Sport Medicine* 2017, **27**(4):e58.
19. Chen Z, Zhao H, Fu N, Chen L: **The diversified function and potential therapy of ectopic olfactory receptors in non-olfactory tissues**. *Journal of cellular physiology* 2018, **233**(3):2104-2115.

20. Moseley ML, Zu T, Ikeda Y, Gao W, Mosemiller AK, Daughters RS, Chen G, Weatherspoon MR, Clark HB, Ebner TJ: **Bidirectional expression of CUG and CAG expansion transcripts and intranuclear polyglutamine inclusions in spinocerebellar ataxia type 8**. *Nature genetics* 2006, **38**(7):758.
21. Chahrour MH, Timothy WY, Lim ET, Ataman B, Coulter ME, Hill RS, Stevens CR, Schubert CR, Greenberg ME, Gabriel SB: **Whole-exome sequencing and homozygosity analysis implicate depolarization-regulated neuronal genes in autism**. *PLoS genetics* 2012, **8**(4):e1002635.
22. Alter MD, Kharkar R, Ramsey KE, Craig DW, Melmed RD, Grebe TA, Bay RC, Ober-Reynolds S, Kirwan J, Jones JJ: **Autism and increased paternal age related changes in global levels of gene expression regulation**. *PloS one* 2011, **6**(2):e16715.
23. Palmieri L, Papaleo V, Porcelli V, Scarcia P, Gaita L, Sacco R, Hager J, Rousseau F, Curatolo P, Manzi B: **Altered calcium homeostasis in autism-spectrum disorders: evidence from biochemical and genetic studies of the mitochondrial aspartate/glutamate carrier AGC1**. *Molecular psychiatry* 2010, **15**(1):38.
24. Izrailev S: **tictoc: Functions for timing R scripts, as well as implementations of Stack and List structures**. *R package version* 2014, **1**.
